# Use of thromboprophylaxis guidelines and risk stratification tools in atrial fibrillation: A survey of general practitioners in Australia

**DOI:** 10.1111/jep.13685

**Published:** 2022-04-06

**Authors:** Eyob Alemayehu Gebreyohannes, Sandra M. Salter, Leanne Chalmers, Jan Radford, Kenneth Lee

**Affiliations:** ^1^ Division of Pharmacy, School of Allied Health The University of Western Australia Perth Western Australia Australia; ^2^ Curtin Medical School Curtin University Perth Western Australia Australia; ^3^ Launceston Clinical School, Tasmanian School of Medicine University of Tasmania Launceston Tasmania Australia

**Keywords:** atrial fibrillation, oral anticoagulants, GPs, general practice, primary care, guideline adherence

## Abstract

**Rationale and Objectives:**

Clinical guidelines produced by cardiology societies (henceforth referred to simply as ‘clinical guidelines’) recommend thromboprophylaxis with oral anticoagulants (OACs) in patients with atrial fibrillation (AF) who have moderate‐to‐high stroke risk. However, deviations from these recommendations are observed, especially in the primary healthcare setting. The primary aims of this study were to evaluate the self‐reported use of AF clinical guidelines and risk stratification tools among Australian general practitioners (GPs), and their perceptions regarding the available resources.

**Method:**

We conducted an online survey of Australian GPs. Descriptive statistics were used to summarise the findings.

**Results:**

Responses from 115 GPs were included for analysis. Respondents reported various ways of accessing thromboprophylaxis‐related information (*n* = 113), including clinical guidelines (13.3%), ‘Therapeutic Guidelines^©^’ (37.2%) and Royal Australian College of General Practitioners websites (16.8%). Of those who reported reasons against accessing information from clinical guidelines (*n* = 97), the most frequent issues were: too many AF guidelines to choose from (34.0%; 33/97), different guidelines for different diseases (32.0%; 31/97), time‐consuming to read guidelines (21.6%; 21/97), disagreements between different guideline recommendations (20.0%; 19/97), conflict with criteria for government subsidy (17.5%; 17/97) and GPs' busy schedules (15.5%; 15/97). When assessing patients' risk of stroke (*n* = 112) and bleeding (*n* = 111), the majority of the respondents reported primarily relying on a formal stroke risk (67.0%) and bleeding risk (55.0%) assessment tools, respectively. Respondents reported using formal stroke and bleeding risk assessment tools mainly when newly initiating patients on therapy (72.4%; 76/105 and 65.3%; 65/101, respectively).

**Conclusion:**

Among our small sample of Australian GPs, most did not access thromboprophylaxis‐related information directly from AF‐specific clinical guidelines developed by cardiology societies. Although the majority reported using formal stroke and bleeding assessment tools, these were typically used on OAC initiation only. More focus is needed on formal risk reassessment as clinically indicated and at regular review.

## 1 INTRODUCTION

Atrial fibrillation (AF) is the most frequently diagnosed cardiac arrhythmia in clinical practice.^1^ According to the Global Burden of Diseases estimates, the global prevalence of AF was 38 million in 2017.^2^ AF is one of the most commonly managed chronic illnesses in general practice in Australia.^3^ It is associated with an increased burden of stroke and systemic embolism.^2^ The risk of stroke associated with AF, especially in the absence of moderate‐to‐severe mitral stenosis or mechanical heart valve, depends mainly on the patient's age and the presence or absence of other commonly concomitant diseases.^4^, ^5^, ^6^

Oral anticoagulants (OACs) are typically used in patients with AF for the prevention of thromboembolic events, mainly stroke. They are associated with a 70% relative reduction in the risk of stroke.^4^ Clinical guidelines produced by cardiology societies worldwide, including the National Heart Foundation of Australia (NHFA) and the Cardiac Society of Australia and New Zealand (CSANZ), recommend thromboprophylaxis with OACs in patients with moderate‐to‐high risk of stroke.^4^, ^5^, ^6^ In addition, assessment of the risk of bleeding is recommended with the aim of identifying and addressing modifiable risk factors for bleeding.^4^, ^5^, ^6^

Thromboprophylaxis that adheres to recommendations based on a formal stroke risk assessment tools such as the CHA_2_DS_2_‐VASc score is associated with better treatment outcomes.^7^ However, deviations from these recommendations, primarily undertreatment, are observed.^8^ Recent Australian studies have reported that 19%–37% of hospitalised AF patients at high risk of stroke did not receive OAC therapy,^9^, ^10^ while the rate of nonprescribing in high‐risk patients in general practices has been reported at 35%–45%.^11^, ^12^

Several factors contribute to OAC undertreatment in AF. Most of the previous studies that reported factors associated with thromboprophylaxis undertreatment in AF were based on findings from retrospective studies and largely outside of the general practice setting.^7^, ^10^, ^13^, ^14^, ^15^ Nonetheless, a previous review article identified that prescriber‐related factors, including their beliefs and practice patterns, were among the major contributors.^8^ Previous qualitative studies reported that more emphasis is given to bleeding than stroke prevention when prescribing OACs, although with limited use of formal bleeding risk assessment tools by prescribers.^16^, ^17^, ^18^ Also, the proportion of prescribers, including general practitioners (GPs, known elsewhere as ‘primary care physicians’), cardiologists and neurologists, who use formal bleeding risk assessment tools seems to correspond with the proportion of those who use formal stroke risk assessment tools.^19^

Uncertainties in prescribers' knowledge and skills in calculating and applying stroke and bleeding risk assessment in AF have been documented elsewhere.^19^ Findings from a study conducted in nine European countries indicate that GPs use different local/national and international guidelines in management of their patients with AF, yet 21% of the small sample of 212 GPs reported not following any specific clinical guidelines.^20^ A recent exploratory qualitative study that conducted semi‐structured interviews among GPs in Western Australia identified the decision‐making process as a key reason for deviations from thromboprophylaxis guidelines by the study participants, with limited use of clinical guidelines and complexities in balancing risk versus benefit of thromboprophylaxis.^16^ In addition to the stroke and bleeding risks, older age, dementia, frailty and falls risk were reported to influence decisions leading to deviations from guideline‐recommended thromboprophylaxis.^16^ Supporting data on where Australian GPs access information regarding thromboprophylaxis in AF and how different factors contribute to their decision‐making process are limited. Therefore, understanding GPs' sources of information upon which they base decisions and the different weights GPs ascribe to various factors in their thromboprophylaxis decision‐making process is important.

Our primary aims were to evaluate the self‐reported use of AF clinical guidelines produced by cardiology societies and risk stratification tools among Australian GPs, and their perceptions regarding the available resources. Our secondary aim was to assess the weightings ascribed by GPs to factors affecting the thromboprophylaxis decision‐making process in patients with AF in Australian general practice.

## 2 METHODS

### 2.1 Study design

We conducted an online survey among GPs practising in general practices in Australia. A questionnaire was developed based on a review of the literature^8^, ^21^ and a previous exploratory qualitative study by the researchers.^16^ The questionnaire contained 3 sections: A sociodemographic section; questions focused on GPs' self‐reported use of clinical guidelines and risk stratification tools in AF; and questions focused on the weight ascribed to different factors in GPs' decision‐making process in prescribing OACs (a scale of −5 to 5, where −5, 0 and 5 indicate highest weight against prescribing OACs, no weight in thromboprophylaxis decisions and highest weight towards prescribing OACs, respectively). We defined clinical guidelines as those that were AF‐specific, which contain thromboprophylaxis‐related recommendations, and were prepared by relevant cardiology societies such as the NHFA/CSANZ, European Society of Cardiology (ESC) and American Heart Association (AHA)/American College of Cardiology (ACC)/Heart Rhythm Society (HRS); these are henceforth referred to simply as ‘clinical guidelines’.^4^, ^5^, ^6^

### 2.2 Procedures and data collection

Before data collection, content validation for the survey tool was completed by four experts (three GPs and one cardiology clinical pharmacist). Then, content validity index for scale (S‐CVI) were calculated in two ways: S‐CVI based on the average method (S‐CVI/Ave) and S‐CVI based on the universal agreement method (S‐CVI/UA).^22^ The survey tool was judged to have good content validity based on the ratings of the four experts (S‐CVI/Ave = 0.98, S‐CVI/UA = 0.93). Suggestions made by the experts were incorporated in the final survey tool.

Multiple strategies were used to recruit respondents, including advertising via professional websites, newsletters and social media (LinkedIn, Twitter and Facebook), and through direct contact, medical practices and professional organisations. In addition, we contracted a commercial company to recruit respondents through a targeted approach (i.e. only those potential respondents who work in healthcare/medical industry). Anonymous data were collected from May 2021 to November 2021 using an online survey management platform, Qualtrics (Qualtrics International Inc.). We followed the Strengthening the Reporting of Observational Studies in Epidemiology (STROBE) statement in reporting this study (Supporting Information).^23^

### 2.3 Eligibility

To take part in the survey, respondents had to be a GP practising in a general practice setting in Australia, and consent to take part in the survey. Respondents who accessed the survey from outside Australia [based on their internet protocol (IP) address], or who only completed the sociodemographic questions, or whose responses were illogical (e.g., respondents with zero years of experiences as a GP; and respondents aged 30–39 years with a 31 years' experience as a GP) were excluded.

### 2.4 Data analysis

Descriptive statistics were used to summarise the findings. Continuous variables were expressed as median and interquartile range (IQR) and categorical variables as frequencies and percentages. A *χ* ^2^ was performed to investigate whether there was association between primarily (i.e., either ‘entirely’ or ‘mainly’) relying on formal stroke risk assessment tools and primarily (i.e., either ‘entirely’ or ‘mainly’) relying on formal bleeding risk assessment tools. All statistical analyses were performed using SAS version 9.4 (SAS institute Inc.).

### 2.5 Ethics

The study was approved by the University of Western Australia Human Research Ethics Committee (RA/4/20/6366).

## 3 RESULTS

### 3.1 Respondents

Responses from 134 survey respondents were recorded. Of these, 19 were excluded because of the following reasons: sociodemographic data only (*n* = 14), illogical responses (*n* = 3) and survey accessed from outside Australia (*n* = 2). The remaining 115 responses were included in the final analyses. Responses were obtained from GPs practising across all States and Territories in Australia with a median (IQR) of 15 (22.0) years' experience as a GP. The age distribution of respondents was representative of the national GP workforce, with the proportion of female respondents (54.8%; 63/115) higher than the national average (47.0%)^24^ (Table 1).

**Table 1:** Respondents' sociodemographic information (*n* = 115)

	*n* (%)
Age in years	
<30	8 (7.0%)
30–39	26 (22.6%)
40–49	31 (27.0%)
50–59	22 (19.1%)
60–70	18 (15.7%)
>70	10 (8.7%)
Gender	
Female	63 (54.8%)
Male	51 (44.4%)
Nonbinary	1 (0.9%)
Median (IQR) years of experience as a GP	15 (22.0)
State or territory where main practice is located	
New South Wales	34 (29.6%)
Queensland	17 (14.8%)
Southern Australia	11 (9.6%)
Victoria	23 (20.0%)
Western Australia	18 (15.7%)
Other	12 (10.4)

Abbreviations: GP, general practitioner; IQR, interquartile range.

### 3.2 Patterns of use of AF thromboprophylaxis guidelines and risk stratification tools

Fifteen (13.3%) respondents reported directly using clinical guidelines to guide thromboprophylaxis prescribing. When asked about frequency of referring to clinical guidelines, one participant did not respond, while the remaining 14 participants reported using clinical guidelines when: managing patients newly diagnosed with AF (50%; 7/14); a clinical decision about anticoagulation is challenging or uncertain (42.9%; 6/14); or a new version of the clinical guideline is available (42.9%; 6/14) (Table 2). Of these respondents (*n* = 15), 14 (93.3%) reported to preferably use the 2018 NHFA/CSANZ guidelines.^4^ The most frequently identified strengths of respondents' preferred clinical guidelines were clear recommendations (60.0%; 9/15), easy to follow algorithms (40.0%; 6/15), detailed recommendations supported by evidence (40.0%; 6/15) and online availability (33.3%; 5/15). Alternatively, the length of the clinical guidelines (20.0%; 3/15) was mentioned as the major limitation (Appendix 1).

**Table 2:** Respondents' use of thromboprophylaxis guidelines in AF

	*n* (%)
Source of information to guide thromboprophylaxis decisions in AF (*n* = 113)	
Directly through clinical guidelines	15 (13.3%)
Therapeutic Guidelines^©^	42 (37.2%)
RACGP websites	19 (16.8%)
Educational sessions (e.g., webinars)	11 (9.7%)
GP CPD websites (e.g., Medcast, Hot Topics, etc.)	9 (8.0%)
Reading of the literature	7 (6.2%)
Other	10 (8.8%)
Frequency of using a guideline (*n* = 14)	
When managing patients newly diagnosed with AF	7 (50.0%)
When a clinical decision about anticoagulation is challenging or uncertain	6 (42.9%)
When a new version of the guideline is available	6 (42.9%)
Every time I manage a patient with AF	1 (7.1%)
Reasons for not using AF clinical guidelines as a primary resource (*n* = 97)	
Too many guidelines to choose from	33 (34.0%)
Too many guidelines for different disease conditions	31 (32.0%)
The guidelines are very long and time‐consuming	21 (21.6%)
The guidelines sometimes disagree with each other	19 (20.0%)
The guidelines sometimes disagree with PBS criteria	17 (17.5%)
My busy schedule	15 (15.5%)
Preference/better familiarity with other options (‘Therapeutic Guidelines^©^’/NPS/GARFIELD tool)	5 (5.2%)
Other	18 (18.6%)

Abbreviations: AF, atrial fibrillation; CPD, continuous professional development; GARFIELD, The Global Anticoagulant Registry in the FIELD; NPS, National Prescribing Service MedicineWise (A not‐for‐profit organisation focused on quality use of medicines in Australia); PBS, Pharmaceutical Benefits Scheme (a government‐funded program that subsidises the cost of medications in Australia); RACGP, The Royal Australian College of General Practitioners (Australia's largest professional general practice organisation).

Ninety‐eight respondents (86.7%) reported that their primary source of thromboprophylaxis‐related information was sources other than clinical guidelines. The most popular source was ‘Therapeutic Guidelines^©^’, an independent and comprehensive source of clinical information covering common disorders seen in clinical practice (37.2%; 42/113).^25^ The most common reasons against using clinical guidelines (*n* = 97) were: too many AF clinical guidelines to choose from (34.0%; 33/97); too many clinical guidelines for different disease conditions (32.0%; 31/97); the very long and time‐consuming nature of reading the clinical guidelines (21.6%; 21/97); disagreements between different clinical guidelines (20.0%; 19/97); conflict with criteria for government subsidy [i.e., the Australian Pharmaceutical Benefits Scheme (PBS)] (17.5%; 17/97); and GPs' busy schedules (15.5%; 15/97) (Table 2).

### 3.3 Stroke and bleeding risk assessment

**Figure 1:**
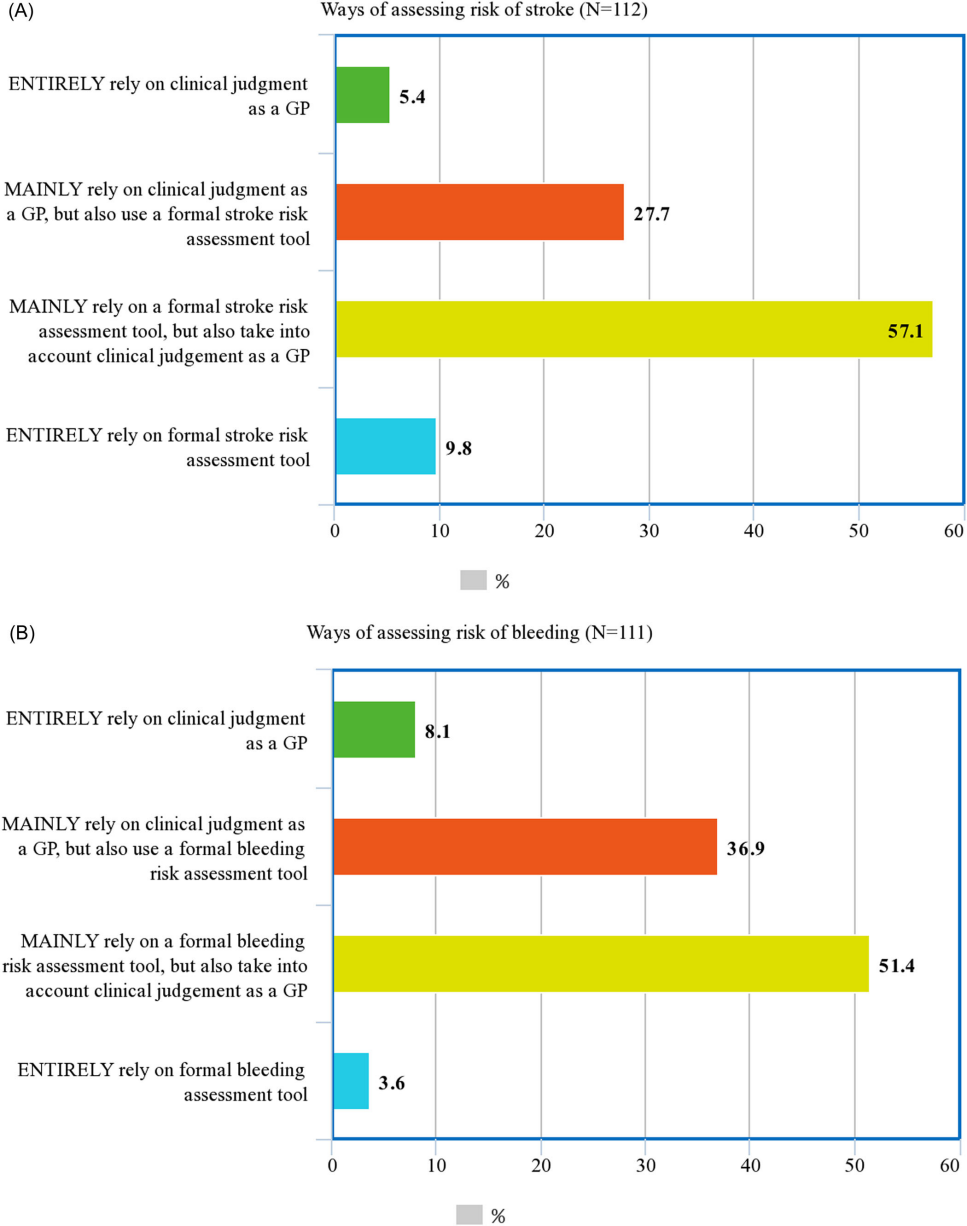
Ways of assessing stroke (A) and bleeding (B) risks. GP, general practitioner

When assessing patients' risk of stroke (*n* = 112; three participants did not respond to this question), the majority (57.1%; 64/112) of the respondents reported mainly relying on a formal stroke risk assessment tool while taking their clinical judgement as a GP into account. Others (27.7%; 31/112) reported they mainly rely on their clinical judgement as a GP while also using formal stroke assessment tools (Figure 1A). Among those who reported using a formal stroke risk assessment tool (*n* = 105; one nonresponse, six reported entirely relying on clinical judgement), the CHA_2_DS_2_‐VASc (73.3%; 77/105), CHA_2_DS_2_‐VA (18.1%; 19/105) and the CHADS_2_ (7.6%; 8/105) were the most preferred. They were mainly used when newly initiating patients on therapy (72.4%; 76/105), whenever a patient's comorbidities change (44.8%; 47/105) and as part of a regular review (27.6%; 29/105) (Table 3).

**Table 3:** Respondents' use of stroke and bleeding risk assessment tools

	*n* (%)
Preferred formal stroke risk assessment tool (*n* = 105)	
CHA_2_DS_2_‐VASc	77 (73.3%)
CHA_2_DS_2_‐VA	19 (18.1%)
CHADS_2_	8 (7.6%)
GARFIELD	1 (1.0%)
Frequency of using the preferred formal stroke risk assessment tool (*n* = 105)	
When newly initiating patients on therapy	76 (72.4%)
Whenever a patient's comorbidities change (e.g. in severity, complications, new comorbidity…)	47 (44.8%)
As part of a regular review (e.g., every 6–12 months)	29 (27.6%)
Every time a patient has a new medication prescribed	16 (15.2%)
Every time the patient visits my office	4 (3.8%)
Other	2 (1.9%)
Preferred formal bleeding risk assessment tool (*n* = 101)	
HAS‐BLED	82 (81.2%)
HEMORR_2_HAGES	10 (9.9%)
ATRIA	6 (5.9%)
ORBIT	3 (3.0%)
Frequency of using the preferred formal bleeding risk assessment tool (*n* = 101)	
When newly initiating patients on OAC therapy	66 (65.3%)
Whenever a patient's comorbidities change (e.g., in severity, complications, new comorbidity…)	35 (34.7%)
As part of a regular review (e.g., every 6–12 months)	24 (23.8%)
Every time a patient has a new medication prescribed	13 (12.9%)
Every time the patient visits my office	5 (5.0%)
Other	6 (5.9%)

Abbreviation: OAC, oral anticoagulant.

When assessing patients' risk of bleeding (*n* = 111; four participants did not respond to this question), half (51.4%; 57/111) of the respondents reported mainly relying on a formal bleeding risk assessment tool while taking their clinical judgement as a GP into account. Others (36.9%; 41/111) reported mainly relying on their clinical judgement as a GP while also using formal bleeding assessment tools (Figure 1B). Among those who reported using a formal bleeding risk assessment tool (*n* = 101; one nonresponse, eight reported entirely relying on clinical judgement), HAS‐BLED was preferred by the majority (81.2%; 82/101); such tools were mainly used when newly initiating patients on OAC therapy (65.3%; 65/101), whenever a patient's comorbidities change (34.7%; 35/101) and as part of a regular review (23.8%; 24/101) (Table 3). There was a positive association between primarily using formal stroke risk and bleeding risk assessment tools, with those who primarily relied on formal tools to assess stroke risk more likely to also rely on formal tools to assess bleeding risk (*χ* ^2^ = 46.1, 1 DF, *p* < 0.001).

### 3.4 The weight of different factors in thromboprophylaxis prescribing

**Figure 2:**
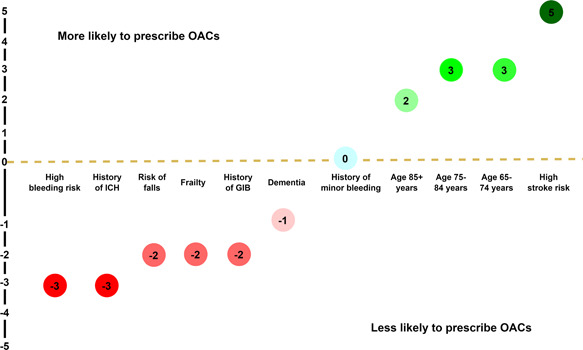
Median of the weight of different factors on thromboprophylaxis decisions (*n* = 114). GIB, gastrointestinal bleeding; ICH, intracranial haemorrhage; OACs, oral anticoagulants

Figure 2 shows the median reported weight of different factors on GPs' decisions to prescribe or not prescribe OACs in patients with AF. While a history of minor bleeding carried negligible weight in OAC prescribing decisions, respondents were more likely to prescribe OACs in patients (in decreasing order of ascribed weight) with a high stroke risk, aged 65–84 years, and aged 85+ years. Respondents were less likely to prescribe OACs (in decreasing order of ascribed weight) where there was a history of intracranial haemorrhage (ICH) or high bleeding risk (equal highest weight), a risk of falls, history of gastrointestinal bleeding (GIB) or frailty, or diagnosed dementia.

## 4 DISCUSSION

Our study provides an important understanding of sources of information used by Australian GPs when making decisions about thromboprophylaxis prescribing in patients with AF. Continuing consistent formal risk assessment remains the main evidence‐to‐practice gap and most respondents in our study reported using formal stroke and bleeding risk assessment tools mainly on OAC initiation only, not consistently across the continuum of care. They also reported accessing thromboprophylaxis‐related information in a variety of ways, but primarily from sources other than cardiology society‐generated clinical guidelines. Further, the patient factors most strongly influencing GPs' prescribing decisions were stroke and bleeding risk, older age and history of ICH. Identifying GPs' sources of prescribing information and their decision‐making process could support efforts to improve guideline usability so that non‐guideline‐adherent OAC prescribing and inconsistent thromboprophylaxis in AF is reduced in Australian general practice.^11^, ^12^ Saying this, we acknowledge that prescribers may appropriately deviate from guideline‐recommended therapy in the interests of person‐centred care, because of individual clinical contexts and the patients' goals of therapy.^16^, ^26^ Documentation of such decisions, for instance with the help of electronic decision supports, could help differentiate situations where patients with AF may benefit from guideline‐adherent therapy from those where deviations from guideline recommendations are more appropriate.^27^

Given that prevention of stroke is the primary indication for OAC prescription in patients with AF and bleeding is recognised as the most significant adverse effect of OACs,^4^ it was not surprising that a high stroke risk and high bleeding risk were ascribed the highest weights when deciding whether to prescribe, or not prescribe OACs, respectively. Therefore, particular attention should be given to the use of formal stroke and bleeding risk assessment tools, as a small number of respondents reported relying solely on clinical judgement to determine these risks. While it was positive that (among respondents who reported using risk assessment tools), contemporary, guideline‐recommended tools (CHA_2_DS_2_‐VASc or CHA_2_DS_2_‐VA and HAS‐BLED) were the preferred stroke and bleeding risk assessment tools, it was concerning that risk assessment, whether stroke or bleeding risk, was only reported as being consistently performed when initiating OACs. Emphasis should be given to the dynamic nature of stroke and bleeding risk, as they change over time because of patients' age and other risk factors.^28^ Recent data from the Australian general practice setting indicated that one‐third of patients whose stroke risk changed from low‐to‐moderate to high were not prescribed OAC therapy. In the remaining patients who received OAC therapy, OAC initiation was reported to be delayed by a median of 2 years, which suggested a need for more frequent stroke risk reassessments.^12^ This is also true with bleeding risk reassessments. In addition to identifying and addressing modifiable bleeding risk factors and reducing bleeding events, more frequent bleeding risk assessment could help with higher prescription of OACs in eligible patients with AF.^29^ The proportion of respondents that reported relying on formal bleeding risk assessment tools as the primary means of assessing patients' risk of bleeding was numerically lower than those who reported using formal stroke risk assessment tools to assess patients' risk of stroke (55.0% vs. 66.9%). This is consistent with previous studies, which have shown that GPs tend to use stroke risk stratification tools more often than they use bleeding risk assessment tools.^8^, ^16^ Importantly, not primarily relying on formal bleeding risk assessment tools was more common among those who also do not primarily rely on formal stroke risk assessment tools. The inconsistent use of stroke and bleeding risk stratification tools means that prescription of OACs in eligible patients may vary among GPs. There is a recognised disparity in OAC prescribing between general practices in Australia: after ranking prescribing rates into quintiles (five equal parts), prescribing in patients with AF who have moderate‐to‐high risk of stroke was 65.6% in the highest practice site quintiles while this figure is only 38.6% in the lowest practice site quintiles.^11^ Therefore, any efforts to improve the use of formal bleeding risk assessment tools should also take improving the use of formal stroke risk assessment tools into consideration. Understanding the reason for these differences may assist in improving the use of OAC prescribing in Australian general practice.

Less than one in six respondents (13.3%) reported that they directly refer to clinical guidelines for recommendations on thromboprophylaxis in AF. Notably, the availability of multiple clinical guidelines for AF, which contained some conflicting recommendations, was a key deterrent to using any clinical guideline. While we did not capture the GPs' perceived areas of conflict between the different AF guidelines, it may be hypothesised that these include recommendations regarding different stroke risk assessment tools (i.e., CHA_2_DS_2_‐VA vs. CHA_2_DS_2_‐VASc). Apart from this notable difference, the most recent versions of the major international AF clinical guidelines have similar recommendations on thromboprophylaxis.^5^, ^6^, ^30^ Hence, the focus should be more on encouraging GPs to make evidence‐based thromboprophylaxis decisions based on their preferred guidelines rather than on selecting a specific clinical guideline. Apart from availability of multiple clinical guidelines for AF, respondents also reported the presence of multiple clinical guidelines for other diseases discouraging the use of any AF guideline. A previous study reported that GPs may be frustrated by the large number of guidelines and perceived associated complications to their patients (e.g., excessive treatment and reduced quality of life) and themselves (e.g., increased insecurity and ‘defensive medicine’ including increased prescribing). In particular, GPs reported feeling compelled to implement guidelines that were not appropriate to their patients' conditions, which present various multimorbidity contexts to manage, when considering recommendations from multiple guidelines.^31^ In addition, our study identified the very long and time‐consuming nature of reading the clinical guidelines, coupled with GPs' busy schedules, as reasons for not using AF clinical guidelines. Previous Australian research identified that GPs preferred shorter guideline formats over longer and more comprehensive formats.^32^ This may be the reason for the preferential use of alternative resources, such as ‘Therapeutic Guidelines^©^’,^25^ over the more comprehensive guidelines produced by cardiology societies.^4^ However, unlike the 2018 NHFA/CSANZ AF guideline,^4^ ‘Therapeutic Guidelines^©^’ lacks recommendations on thromboprophylaxis prescribing in patients who are at risk of falls, are frail, or have a history of ICH or GIB, that is, the factors ascribed the highest weights when deciding not to prescribe OACs.^25^ One possible approach to addressing the issues identified in this study with the use of the current clinical guidelines would be a focused codesign process with GPs, to develop setting‐specific guidelines that are more fit for purpose within Australian general practice—namely, shorter, consistent with practical clinical considerations, more cognisant of complex multimorbidity, and addressing the major barriers to GPs' prescribing of OACs, such as falls, frailty and history of ICH or GIB.

In Australia, the cost of medications are subsidised by the PBS, a government‐funded program based on medications' efficacy, safety and cost‐effectiveness.^33^ One important challenge raised by 17.5% of the respondents was disagreements between the recommendations of the guidelines and the PBS criteria. Disagreements between guideline recommendations and the PBS criteria were also identified in our recent qualitative study.^16^ This is an important practical challenge to GPs as some of the patients who are eligible for oral anticoagulation based on the CHA_2_DS_2_‐VA score may not be eligible under the PBS criteria, which uses the CHADS_2_ score.^34^, ^35^, ^36^ A previous Australian study reported large differences in the proportion of patients with AF who were classified high‐risk for stroke depending on the risk stratification tool used.^37^ Even though the aim of the PBS criteria is not to make clinical decisions on whether a patient with AF is eligible for anticoagulation, the fact that the cost of the prescribed OAC depends on eligibility can affect thromboprophylaxis decisions. Therefore, updating the PBS criteria to be in line with the guideline recommendations is warranted to minimise another potential source of confusion for GPs.

### 4.1 Strengths and limitations

Interpretation of the findings of the study should consider the potential limitations of this study. Considering more than 37,000 GPs practise across Australia,^24^ the sample size was small making any generalisations and inferences difficult. Also, because this was an anonymous, online survey, it was not possible to calculate the response rate of respondents, as the denominator was not able to be determined. Despite these limitations, the study provides insight into sources of information used by GPs when prescribing thromboprophylaxis in AF, their reasons for not accessing such information from AF clinical guidelines, and the weight they give to different important factors in their thromboprophylaxis decision‐making, which may prove useful in developing future strategies to ensure consistent, high‐quality thromboprophylaxis for all Australians with AF.

## 5 CONCLUSIONS

Most respondents among this small sample of Australian GPs access thromboprophylaxis‐related information in AF from sources other than clinical guidelines produced by cardiology societies, most of which lacked advice on prescribing in complex comorbid clinical cases. Strategies are required to address the lack of usability of current guidelines, including too many AF clinical guidelines that are often too long, disagreements between different guideline recommendations, and inconsistencies with clinical guidelines for other comorbid diseases. Ensuring government‐funding criteria matches clinical guideline recommendations may provide clarity in prescribing and simplify the use of clinical guidelines. Although the majority of respondents focussed strongly on stroke and bleeding risk in making prescribing decision and used formal risk assessment tools, these were typically used on OAC initiation only; future work is needed to promote formal review on an ongoing basis.

## AUTHOR CONTRIBUTIONS

**Eyob Alemayehu Gebreyohannes**: concept and design; literature search; questionnaire validation; participant recruitment; data analysis; writing the first draft of the manuscript; critical revision of the manuscript; and final approval of the submitted manuscript. **Sandra Salter**, **Leanne Chalmers**, **Jan Radford** and **Kenneth Lee**: concept and design; participant recruitment; critical revision of the manuscript; and final approval of the submitted manuscript.

## CONFLICTS OF INTEREST

The authors declare no conflicts of interest.

## ETHICS STATEMENT

The study was approved by the University of Western Australia Human Research Ethics Committee (RA/4/20/6366).

## Data Availability

The data that support the findings of this study are available on request from the corresponding author. The data are not publicly available due to ethical restrictions.

## APPENDIX A

Table A1

**Table A1:** Strengths and limitations of routinely used thromboprophylaxis guidelines in AF (*n* = 15)

	*n* (%)
Strengths of clinical guidelines	
Clear recommendations	9 (60.0%)
Detailed recommendations supported by evidence	6 (40.0%)
Easy to follow algorithms	6 (40.0%)
Online availability	5 (33.3%)
Clinical applicability/flexibility	3 (20.0%)
Concise	3 (20.0%)
Most authoritative guideline in Australia	1 (6.7%)
Major limitations of clinical guidelines	
I have not noticed any major limitations.	9 (60.0%)
Too long	3 (20.0%)
Difficult to access/not user‐friendly	2 (13.3%)
Disagrees with the PBS criteria	1 (6.7%)
Do not consider patient preferences	1 (6.7%)
Limited clinical flexibility (not patient‐specific)	1 (6.7%)
Unclear recommendations	1 (6.7%)
Difficult to follow algorithms	1 (6.7%)
Helpfulness of clinical guidelines in challenging/uncertain clinical decisions	
Very helpful	3 (20.0%)
Helpful	8 (53.3%)
Slightly helpful	4 (26.7%)
Not helpful at all	0 (0.0%)

Abbreviations: AF, atrial fibrillation; PBS, Pharmaceutical Benefits Scheme.
